# Cost-Effectiveness of Semaglutide 2.4 mg for Obesity Disease Management in Japan: A Lifetime Economic Modeling Study Incorporating Retreatment Scenarios

**DOI:** 10.36469/001c.162614

**Published:** 2026-06-30

**Authors:** Yuta Kamada, Shogo Wada, Hiroyuki Matsuda, Yawen Dai

**Affiliations:** 1 Novo Nordisk Pharma Ltd., Tokyo, Japan; 2 IQVIA Solutions Japan G.K., Tokyo, Japan

**Keywords:** Japan, diabetes, health technology assessment, cost-effectiveness analysis, core obesity model, obesity, semaglutide

## Abstract

**Background:**

Obesity is a chronic disease associated with substantial morbidity and healthcare costs. In Japan, the Optimal Use Promotion Guidelines (OUG) impose restrictions on patient eligibility for semaglutide 2.4 mg and limit treatment duration. This study evaluated the cost-effectiveness of semaglutide 2.4 mg in Japan under retreatment assumptions informed by OUG requirements.

**Methods:**

A Markov cohort model (Core Obesity Model) was used to evaluate lifetime clinical and economic outcomes from a Japanese public payer perspective. The Core Obesity Model assesses the associations between risk factors and the incidence of obesity-related complications. People with obesity disease and either a BMI ≥35 kg/m2 with at least 1 obesity-related comorbidity (hypertension, dyslipidemia, or type 2 diabetes), or a BMI ≥27 kg/m² with at least 2 obesity-related health disorders, were included. The intervention was semaglutide 2.4 mg plus diet and exercise vs diet and exercise alone. We modeled retreatment scenarios (0, 1, or 2 retreatments) with a fixed 1-year off-treatment period after each regimen. Complication costs were sourced from Japanese studies. The primary outcome was the incremental cost-effectiveness ratio (ICER) in Japanese yen per quality adjusted life year (QALY), discounted at 2%.

**Results:**

In the base case, semaglutide 2.4 mg reduced the incidence of obesity-related complications, such as type 2 diabetes (T2D), cardiovascular events, obstructive sleep apnea, and gout. The ICER was ¥5 300 580/QALY for patients with obesity disease without T2D, and ¥7 077 984/QALY for patients with obesity disease with T2D. Retreatment significantly improved QALY gains compared with limited or no retreatment, reflecting longer maintenance of treatment benefit.

**Conclusions:**

Accounting for OUG consistent retreatment in Japan improved the cost-effectiveness of semaglutide 2.4 mg and provided a more realistic representation of long-term obesity management than single course assumptions. Analyses that omit retreatment may underestimate its long-term value in Japanese clinical practice. Results were broadly robust, although uncertainty remains around retreatment efficacy and long-term discontinuation. Semaglutide 2.4 mg represents a clinically and economically valuable intervention for obesity disease in Japan. These findings emphasize the need for long‑term, clinically realistic assessments when evaluating obesity treatments in Japan.

## BACKGROUND

Obesity disease is an increasing public health challenge in Japan, where it is defined as body mass index (BMI) ≥25 kg/m² and is associated with a substantial burden of obesity-related comorbidities, including type 2 diabetes (T2D), cardiovascular disease (CVD), and impaired quality of life.^1,2^ According to the 2023 National Health and Nutrition Survey (NHNS), the prevalence of obesity (BMI ≥25 kg/m²) was 26% among people aged ≥15 years, with noticeable sex differences (31.5% in men and 21.1% in women).^3^ Although Japan has a comparatively low prevalence under the World Health Organization definition (BMI ≥30 kg/m²), NHNS data still indicates nontrivial proportions of the population still meet this threshold (eg, 4.5% overall among those aged ≥15 years). A targeted review of the Japanese evidence base has demonstrated that obesity is consistently associated with increased risks of complications and meaningful impairments in both physical and mental quality of life.^4^ Importantly, recent nationwide health check-up data in Japan (2015-2020; n = 8 155 894 adults aged 35-69 years) demonstrate an upward BMI trend, with positive annual mean BMI changes across all sex and age subgroups, and larger increases among younger and middle-aged adults.^5^ This epidemiological and clinical burden translates into substantial economic impact. Using a public economic framework, Igarashi et al estimated that overweight/obesity generates an annual fiscal burden of approximately US $13.41 billion (¥1925 billion)—around 0.4% of Japan’s GDP—driven by excess healthcare expenditures, reduced employment and income, increased sick leave, and higher government transfers, including pensions associated with early retirement.^6^

Given the magnitude and persistence of this burden, effective treatment strategies are essential to prevent long-term complications and mitigate downstream costs. According to the Japanese Guidelines for the Management of Obesity Disease 2022, first-line treatment consists of lifestyle interventions such as dietary modification and exercise therapy. When these interventions fail to achieve adequate weight reduction, pharmacological therapy aimed at weight loss is recommended as a second-line treatment for individuals.^7^

Semaglutide 2.4 mg, a glucagon-like peptide-1 receptor agonist, has demonstrated superior efficacy in weight reduction and cardiovascular risk mitigation in the global phase 3 Semaglutide Treatment Effect in People with Obesity (STEP) program. In the phase III STEP 6 trial conducted in Japan and South Korea, semaglutide 2.4 mg resulted in a mean percentage change in body weight from baseline to week 68 of -13.2% (standard error [SE] 0.5), compared with -2.1% (SE 0.8) achieved with placebo, corresponding to a between-group difference of -11.1% (95% confidence interval [CI], -12.9 to -9.2) using the treatment policy estimand.^8^

Treatment eligibility includes adults with obesity disease who have a BMI ≥35 kg/m² and at least 1 of the following conditions— hypertension, dyslipidemia, or type 2 diabetes—or a BMI ≥27 kg/m² with at least 2 obesity-related health disorders. Semaglutide 2.4 mg is subject to specific restrictions in Japan.^9^ It is indicated only for patients with obesity disease who have shown insufficient response after at least 6 months of lifestyle interventions (diet and exercise [D&E]). In addition, treatment duration is limited to a maximum of 68 weeks per course. If obesity worsens after treatment discontinuation, retreatment is generally permitted only after the patient has undergone at least 6 months of an off-treatment period of structured lifestyle interventions equivalent to those required at treatment initiation. In exceptional cases where obesity-related health conditions deteriorate, earlier retreatment may be allowed prior to completion of the 6-month period, provided that clinicians carefully assess clinical necessity and establish an appropriate treatment plan.

The economic value of semaglutide 2.4 mg may have been underestimated in previous cost-effectiveness analyses (CEA) conducted in Japan by Center for Outcomes Research and Economic Evaluation for Health (C2H), a health technology assessment (HTA) organization.^10^ A key limitation was the handling of treatment discontinuation and potential retreatment under Japan’s OUG. Given the chronic nature of obesity disease and common occurrence of weight regain after discontinuation of GLP-1 receptor agonist therapy, retreatment is clinically plausible and supported by real-world evidence.^11^ However, the cost effectiveness of retreatment remains uncertain; therefore, CEAs limited to a single treatment course may underestimate the long-term value of therapy in Japan.

The objective of this study is to evaluate the cost-effectiveness of semaglutide 2.4 mg in Japan using a lifetime horizon that explicitly incorporates retreatment scenarios to better reflect the long-term economic value in chronic obesity management.

## METHODS

### Study Design and Model Analysis

**Figure 1.**
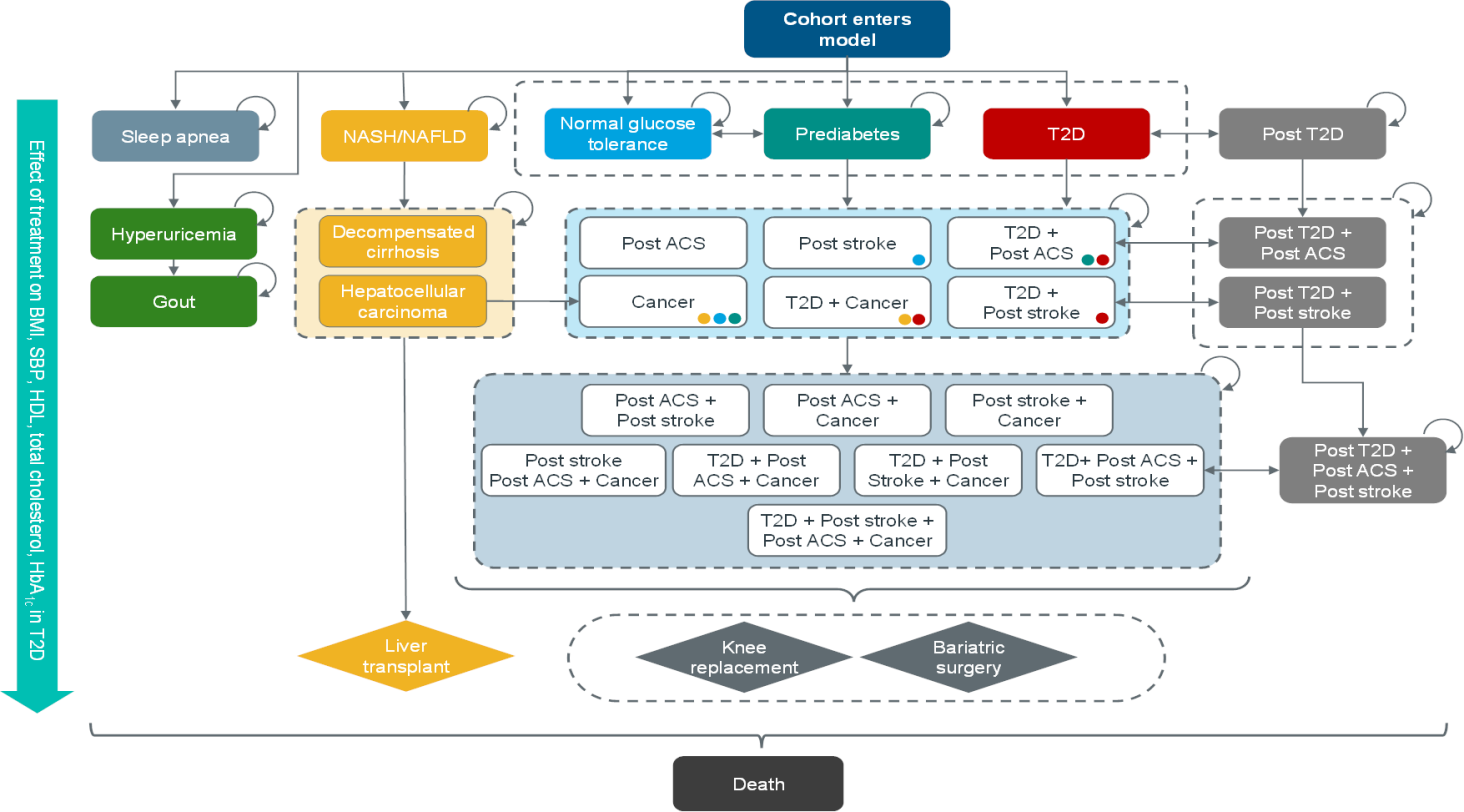
Model Structure Schematic Reproduced/adapted from Lopes (2021)^12^ with permission from Wiley.

We utilized the Core Obesity Model (COM), a validated Markov cohort model, to simulate the long-term clinical and economic outcomes of obesity disease management (**Figure 1**).^12^ Model structure, health states, risk-equation implementation, cycle conventions (quarterly cycles in Year 1, annual cycles thereafter), and half-cycle correction followed published COM descriptions and external validations.^13^

A lifetime horizon was applied to capture the long-term development of obesity-related complications and associated mortality. The primary outcome was the incremental cost-effectiveness ratio (ICER) (¥/QALY). Secondary outcomes included ICER differences across retreatment scenarios and cost breakdowns. Both costs and health outcomes (QALYs) were discounted at an annual rate of 2%, consistent with Japanese health economic evaluation guidelines.^14^ The analysis was conducted from the perspective of the Japanese public healthcare payer.

In Japan, semaglutide 2.4 mg underwent a cost-effectiveness evaluation within the national HTA framework to inform evidence-based policy after reimbursement listing.^10^ The present study adopted the same modeling perspective and population structure but introduced the following 3 predefined modifications to better reflect realistic long-term obesity management pathways:

1. **Retreatment module:** A new module was incorporated to allow the assessment of clinical and economic outcomes associated with re-initiating semaglutide following treatment discontinuation.
2. **Updated drug prices:** Drug acquisition costs were updated to reflect the most recent National Health Insurance (NHI) prices.
3. **Revised discontinuation assumptions:** The discontinuation structure from the original CEA model was replaced with assumptions intended to more closely reflect treatment-continuation patterns observed in Japanese clinical practice. These assumptions were reviewed and validated by a clinician specializing in obesity who is a board member of the Japanese Society for the Study of Obesity and has expertise in endocrinology and metabolism in Japan.

The revised approach to modeling discontinuation is described in detail below.

### Population and Eligibility

The modeled population comprised people with obesity disease, stratified by baseline T2D status (with non-T2D vs T2D; non-T2D was defined as normal glucose tolerance or prediabetes at baseline), consistent with the C2H CEA. Baseline characteristics (eg, age, sex, systolic blood pressure [SBP], lipids, glycemic status distribution) were aligned with the Japanese participants in the STEP 6 trial, except for BMI (**Table 1**). A BMI of 35 kg/m^2^ was applied to better reflect contemporary Japanese clinical practice for semaglutide candidates with the guideline definition of severe obesity disease.^2^ All baseline characteristics were reviewed and validated by the same clinician described above.

**Table 1.** Cohort Characteristics at Baseline, Weighted by Glycemic Status: Non-T2D + T2D

	**Non-T2D, Mean**	**T2D, Mean**
Age (years)	50	53
Body mass index (kg/m^2^)	35	35
Height (m)	1.66	1.65
SBP (mmHg)	134	134
Total cholesterol (mg/dL)	208	191
HDL cholesterol (mg/dL)	51.4	51.7
HbA1c (%)	5.8	8.0
T2D duration (years)	0	7.8
Triglycerides (mg/dL)	148	156
Smokers (%)	20.5	20.2
Females (%)	37.1	36.4

Abbreviations: HbA1c, glycated hemoglobin; HDL, high-density lipoprotein; SBP, systolic blood pressure; T2D, type 2 diabetes.

### Interventions and Comparators

The intervention arm received semaglutide 2.4 mg administered once weekly as an adjunct to D&E under the national health insurance system. This arm is referred to as the “semaglutide 2.4 mg” arm. The comparator arm received D&E alone, referred to as the D&E arm.

To reflect the chronic and relapsing nature of obesity disease, the following 3 retreatment scenarios were evaluated. Because the OUG specifies a maximum course duration of 68 weeks but COM operates on annual cycles, each course was operationalized as a 1-year treatment period. Moreover, while the OUG defines an off-treatment period as at least 6 months, we defined it as 1 year, taking into account the rate of increase in BMI and other laboratory values after treatment discontinuation, as well as clinical expert opinion. Retreatment scenarios are defined as follows:

1. **No retreatment:** Treatment is limited to a single course, consistent with assumptions used in previous Japanese CEA reports.
2. **One retreatment:** Patients receive 1 additional course of semaglutide following an off-treatment period.
3. **Two retreatments (base case):** Patients receive up to 2 additional courses, reflecting a more realistic long-term obesity disease management approach.

### Model Inputs and Setting

**Treatment effects, parameter trajectories, costs and utility:** Treatment effects (weight loss percentage, SBP, lipids, and glycemic status) were sourced from the STEP 6 trial (Japan/Korea cohort) and the broader STEP program. Short-term effects at 28 and 68 weeks were implemented using the trial-informed ratio method used in COM, a timepoint scaling factor used to preserve the empirically observed trajectory of treatment effects such as weight change between weeks 28 and 68. Post-treatment catch-up was modeled to return risk factors toward baseline or toward their natural trajectory under D&E, consistent with established COM precedents (**Table 2**).^13,15^

**Table 2.** Parameters – Changes in Body Weight, Systolic Blood Pressure, Total Cholesterol, HDL, HbA1c, and Prediabetes Reversal vs Baseline by Glycemic Status Subgroup in STEP 6 trial (FAS)

	**Semaglutide 2.4 mg, Mean**	**D&E FAS, Mean**
Change in body weight (%)		
Subgroup: Non-T2D at baseline		
Cycles 2 and 3	-8.99	−2.61
Cycle 4	−14.49	−2.35
Subgroup: T2D at baseline		
Cycles 2 and 3	−5.88	−1.55
Cycle 4	−9.27	−1.60
Changes in SBP (mmHg)		
Subgroup: Non-T2D at baseline		
Cycles 2 and 3	−11.13	−5.90
Cycle 4	−11.12	−6.27
Subgroup: T2D at baseline		
Cycles 2 and 3	−3.00	−3.00
Cycle 4	−10.06	−2.28
Changes in total cholesterol (mg/dL)		
Subgroup: Non-T2D at baseline		
Cycles 2 and 3	−25.01	−6.57
Cycle 4	−16.43	−0.37
Subgroup: T2D at baseline		
Cycles 2 and 3	−22.82	4.02
Cycle 4	−18.94	7.21
Change in HDL cholesterol (mg/dL)		
Subgroup: Non-T2D at baseline		
Cycles 2 and 3	−1.14	3.02
Cycle 4	3.14	3.14
Subgroup: T2D at baseline		
Cycles 2 and 3	−0.94	2.81
Cycle 4	3.49	3.49
Change in HbA1c (%)		
Subgroup: Non-T2D at baseline		
Cycles 2 and 3	0.00	0.00
Cycle 4	0.00	0.00
Subgroup: T2D at baseline		
Cycles 2 and 3	−2.11	−0.26
Cycle 4	−2.14	0.17
Prediabetes reversal from prediabetes to normal glucose tolerance (%)		
Subgroup: Non-T2D at baseline		
Cycles 2	90.70	32.00
Severe gastrointestinal adverse events (%)		
Cycles 1-4	1.30	0.38
Cycles 6, 8	5.08	1.52

Abbreviations: D&E, diet and exercise; FAS, full analysis set; HbA1c, glycated hemoglobin; HDL, high-density lipoprotein; SBP, systolic blood pressure; T2D, type 2 diabetes.

Treatment effects were applied in each model cycle and maintained while patients were on treatment. After treatment discontinuation, effects were assumed to wane gradually over a 3-year catch-up period.^16^ Natural BMI progression beyond this period followed annual BMI increases of 0.1447 kg/m^2^ (males) and 0.1747 kg/m^2^ (females) for non-T2D and 0.0398 kg/m^2^ (males and females) for T2D, based on data from Ara et al.^17^ For patients with T2D, HbA1c progression was drawn from the United Kingdom Prospective Diabetes Study Outcomes Model (UKPDS 68).^18^

Baseline (complication-free) utilities were estimated using a multivariable regression model informed by nationally representative, patient level data from the 2017 and 2018 Health Survey for England.^19^ The EQ-5D utilities were subsequently regressed on polynomial BMI terms (linear, quadratic, and cubic), while adjusting for demographic and socioeconomic characteristics, lifestyle behaviors, and obesity-related comorbidities. This approach allowed complication-free utility to vary continuously with BMI over time, with BMI-related disutility derived endogenously from the estimated regression coefficients rather than sourced as fixed external decrements. Health state utilities for conditions such as T2D, post–acute coronary syndrome, and obstructive sleep apnea, and adverse event disutilities, were obtained from the published literature. Japanese-specific utilities were used when available (**Supplementary Table S1**).

Costs included drug acquisition and administration, using current NHI prices. Complication costs associated with major obesity-related complications such as stroke, myocardial infarction, and ongoing treatment for type 2 diabetes were sourced from analyses of Japanese real-world administrative claims data (IQVIA Claims data) reported in the previous CEA (**Supplementary Table S1**).

**Retreatment assumptions**: Because no dedicated clinical trials or real-world evidence currently characterize the effects of semaglutide retreatment globally, we specified a set of assumptions for implementing retreatment within the COM framework. The treatment effect of each retreatment course was assumed to be equivalent to that of the initial treatment course. When retreatment effects produced clinical values (eg, BMI, HbA1c, or SBP) that exceeded plausible ranges for the general patient population, effect sizes were capped at the minimum or maximum effect observed after the initial administration. All modeling assumptions were reviewed and validated by the same clinical expert described above and supported the clinical plausibility in Japanese practice.

**Dropout modeling**: Long-term discontinuation of D&E was modeled by extrapolating dropout rates reported in Ohira et al^20^ and by treating the higher post-discontinuation dropout rate in the semaglutide arm to reflect observed differences in treatment persistence between the two arms. Under this attenuation rule, the dropout rates of the D&E group and the semaglutide group were assumed to converge within 1 to 10 years after stopping semaglutide.^10^

However, this assumption resulted in implausibly rapid worsening of BMI and other clinical outcomes after semaglutide discontinuation. Consequently, the rate of increase (ie, deterioration) in BMI and other outcomes in the semaglutide group became greater than that of the comparator D&E group. To address these concerns, the following adjustments were implemented.

In the present analysis, treatment discontinuation was conceptualized as a behavioral process that becomes independent of prior pharmacotherapy once semaglutide has been stopped. Individuals who discontinued semaglutide and remained on D&E were assumed to have the same probability of discontinuing D&E as those who had been receiving D&E alone. Thus, no persistent adherence advantage was assumed after discontinuation of semaglutide. Based on this behavioral parity principle, arm-specific discontinuation rates were recalculated so that the post-semaglutide discontinuation probability of stopping D&E after discontinuing semaglutide was identical to that of patients in the D&E group.

During retreatment periods, discontinuation was modeled using these recalculated rates. To avoid introducing additional uncertainty, no incremental effect of retreatment on discontinuation was assumed. Retreatment did not alter the underlying probability of stopping D&E. The revised dropout rates were reviewed and validated by the same clinical expert described above, who supported their clinical plausibility in Japanese practice.

**Risk equations and outcomes mapping**: Transitions for incident and recurrent cardiovascular events, T2D, sleep apnea, knee replacement (osteoarthritis), and cancers (colon, postmenopausal breast, postmenopausal endometrial) followed the validated set of Asia/Japan-specific risk equations embedded in COM,^13^ consistent with the C2H semaglutide analysis.^10^

Except for the retreatment and dropout assumptions described above, all clinical and structural components, including those not described in detail for brevity (eg, mortality, utility/decrement), are fully aligned with the previous CEA.^10^

### Sensitivity Analyses

Scenario analyses explored how alternative retreatment policies might influence the cost-effectiveness results. These scenarios ranged from no additional treatment courses to more permissive approaches that allow reinitiation of semaglutide after an off-treatment period.

Deterministic sensitivity analyses (DSA) were conducted by varying key model parameters within plausible ranges. When a 95% confidence interval (CI) was available, the CI bounds were used; when unavailable, a ±20% range around the base-case value was applied.

Probabilistic sensitivity analyses (PSA) were performed using 1000 Monte Carlo iterations, with model parameters sampled from their respective probability distributions as implemented in COM. Results were presented as a cost-effectiveness acceptability curve.

## RESULTS

### Base Case Analysis (2 Retreatments)

In the base-case analysis with 2 retreatments, semaglutide 2.4 mg plus D&E incurred higher lifetime costs but generated meaningful health gains compared with D&E alone. In the non-T2D cohort, total costs were ¥7 595 634 compared with ¥6 926 474 for the comparator, with incremental gains of 0.126 QALYs and 0.044 life-years, resulting in an ICER of ¥5 300 580/QALY. In the T2D cohort, total costs were ¥10 838 272 compared with ¥9 805 535, with incremental gains of 0.146 QALYs and 0.077 life-years, yielding an ICER of ¥7 077 984/QALY (**Table 3**).

**Table 3.** Base Case Cost-Effectiveness Results

	**Obesity Treatment Costs (¥)**	**Total Cost (¥)**	**Total QALY**	**Incremental Cost (¥)**	**Incremental QALY**	**ICER (¥/QALY)**
Subgroup: Non-T2D at baseline						
Diet and exercise	0	6 926 474	16.048	–	–	–
Semaglutide 2.4 mg	734 246	7 595 634	16.174	669 160	0.126	5 300 580
Subgroup: T2D at baseline						
Diet and exercise	0	9 805 535	14.304	–	–	–
Semaglutide 2.4 mg	1 047 903	10 838 272	14.450	1 032 737	0.146	7 077 984

Abbreviations: ICER, incremental cost-effectiveness ratio; QALY, quality-adjusted life-year.

### Clinical Outcomes

Over the lifetime horizon, semaglutide 2.4 mg was projected to decrease time spent in clinically meaningful health states representing obesity-related complications (**Table 4**). In the non-T2D cohort, time without complications increased by 55.354 patient-years per 100 persons (semaglutide, 1082.935; D&E, 1027.581), while time spent in prediabetes decreased by 23.678 (semaglutide, 213.490; D&E, 237.168), and time with T2D decreased by 25.963 patient-years (semaglutide, 1248.796; D&E, 1274.759). In the T2D cohort, semaglutide 2.4 mg similarly reduced the incidence of acute cardiovascular events and decreased time spent in costly post-event states, such as post-ACS, post-stroke, and gout. This indicates that semaglutide 2.4 mg not only slows metabolic deterioration but also reduces exposure to high-cost complications, explaining the observed cost offsets despite higher drug expenditures.

**Table 4.** Breakdown of Clinical Results

	**Subgroup: Non-T2D Cohort at Baseline**			**Subgroup: T2D Cohort at Baseline**		
**Semaglutide 2.4 mg**	**D&E**	**Incremental Results**	**Semaglutide 2.4 mg**	**D&E**	**Incremental Results**	
Event rate per 100 patient-years						
CV events	3.737	3.784	-0.047	4.298	4.362	−0.063
Knee replacement	0.805	0.806	−0.001	0.896	0.896	0.000
Cumulative health state occupancy per 100 patients (undiscounted)						
No comorbidity	1082.935	1027.581	55.354	0.000	0.000	0.000
OSA	1397.081	1408.522	−11.441	1306.707	1308.340	−1.633
Prediabetes	213.490	237.168	−23.678	0.000	0.000	0.000
T2D	1248.796	1274.759	−25.963	2601.526	2590.714	10.812
Post-ACS	220.899	222.840	−1.941	242.624	245.900	−3.276
Cancer	59.639	60.561	−0.922	46.842	47.856	−1.014
Post-stroke	720.168	727.085	−6.917	778.006	789.327	−11.321
Gout	567.259	581.921	−14.662	435.785	456.132	−20.347
Mean LY and QALYs per patient						
Undiscounted LY	28.360	28.295	0.065	26.015	25.907	0.108
Undiscounted QALYs	21.125	20.969	0.156	18.615	18.435	0.180
Discounted LY	21.278	21.235	0.044	19.864	19.787	0.077
Discounted QALYs	16.174	16.048	0.126	14.450	14.304	0.146
QALY breakdown by comorbidity (discounted) per patient						
No comorbidity	7.823	7.339	0.484	0.000	0.000	0.000
OSA	−0.488	−0.494	0.006	−0.468	−0.469	0.001
Prediabetes	−0.045	−0.046	0.001	−0.035	−0.037	0.002
T2D	1.603	1.814	−0.211	0.000	0.000	0.000
Post-ACS	4.377	4.508	−0.131	12.143	11.955	0.188
Cancer	0.522	0.524	−0.002	0.420	0.426	−0.006
Post-stroke	0.207	0.211	−0.003	0.118	0.121	−0.003
Gout	2.338	2.357	−0.019	2.452	2.492	−0.039

Abbreviations: ACS, acute coronary syndrome; CV, cardiovascular; D&E, diet and exercise; OSA, obstructive sleep apnea; QALY, quality-adjusted life-year; T2D, type 2 diabetes.

### Scenario Analysis: Impact of Retreatment Policy

Restricting retreatment was shown to increase ICERs by shortening time on effective therapy and reducing long-term QALY gains (**Table 5**). Relative to the base case (2 retreatments; non-T2D ¥5 300 580/QALY; T2D ¥7 077 984/QALY), no retreatment (Scenario 1, initial treatment course only) yielded ¥6 131 873/QALY (non-T2D) and ¥11 015 920/QALY (T2D). Allowing 1 retreatment (Scenario 2, 2 treatments; initial treatment and retreatment) improved cost-effectiveness but it remained less favorable than the base case (¥5 708 582/QALY and ¥8 143 283/QALY, respectively).

**Table 5.** Scenario Analyses (Costs in 2025 ¥)

	**Obesity Treatment Costs (¥)**	**Total Cost (¥)**	**Total QALY**	**Incremental Cost (¥)**	**Incremental QALY**	**ICER (¥/QALY)**
Scenario 1: One semaglutide 2.4 mg treatment						
Subgroup: Non-T2D at baseline						
Diet and exercise	0	6 926 474	16.048	–	–	–
Semaglutide 2.4 mg	339 757	7 263 308	16.103	336 834	0.055	6 131 873
Subgroup: T2D at baseline						
Diet and exercise	0	9 805 535	14.304	–	–	–
Semaglutide 2.4 mg	395 344	10 214 879	14.341	409 343	0.037	11 015 920
Scenario 2: Two semaglutide 2.4 mg treatments						
Subgroup: Non-T2D at baseline						
Diet and exercise	0	6 926 474	16.048	–	–	–
Semaglutide 2.4 mg	568 105	7 457 046	16.141	530 572	0.093	5 708 582
Subgroup: T2D at baseline						
Diet and exercise	0	9 805 535	14.304	–	–	–
Semaglutide 2.4 mg	744 032	10 548 481	14.395	742 946	0.091	8 143 283

Abbreviations: ICER, incremental cost-effectiveness ratio; QALY, quality-adjusted life-year.

### Sensitivity Analysis

DSA identified the discount rate on health benefits as the key ICER driver (**Supplementary Figure S1**). In the non-T2D group, the ICER values ranged approximately from ¥4 295 917 to ¥6 290 428/QALY. Weight loss–related parameters, including D&E-arm weight reduction, semaglutide weight reduction, and baseline BMI, formed the second most influential drivers, with ICERs largely between ¥4 902 733 and ¥5 766 979/QALY. Other parameters, such as natural weight regain, event disutilities, and baseline CVD prevalence, had smaller impacts.

A similar pattern was observed in the T2D cohort but at higher ICER levels. The discount rate for benefits again showed the greatest impact (¥5 741 141-¥8 438 964/QALY), followed by baseline BMI and semaglutide-related weight-loss parameters (range, ¥6 420 657-¥7 941 372/QALY). Other clinical and utility inputs (eg, baseline CVD prevalence, stroke disutility, D&E-arm weight loss) produced modest changes relative to the main drivers.

PSA indicated that semaglutide 2.4 mg plus D&E showed a high probability of being cost-effective at a ¥7 500 000/QALY threshold, with probabilities of 100% in the non-T2D cohort and 80% in the T2D cohort (**Supplementary Figure S2**). At a ¥10 000 000/QALY threshold, the probability reached 100% in both cohorts.

## DISCUSSION

In the base-case analysis assuming two retreatments with semaglutide 2.4 mg, the ICER was ¥5 300 580/QALY in the non-T2D population and ¥7 077 984/QALY in the T2D population. These ICERs corresponded to lifetime incremental costs of ¥669 160 and ¥1 032 737 per patient, and incremental QALYs of 0.126 and 0.146 per patient, respectively. Overall, these findings suggest that semaglutide 2.4 mg would likely be considered cost-effective within the Japanese evaluation context, while providing clinically meaningful long-term health gains. Importantly, cost-effectiveness improved when retreatment was included. Compared with a single treatment course (no retreatment), increasing retreatments consistently reduced the ICERs in both the non-T2D and T2D cohorts, indicating that retreatment enhances long-term value. The base-case results assuming 2 retreatments demonstrated better cost-effectiveness than the C2H results (no retreatment), which represent the cost-effectiveness analysis of semaglutide 2.4 mg in Japan (C2H non-T2D, ¥13 095 027/QALY; T2D, ¥18 216 862/QALY).^10^

Beyond economic endpoints, the model also showed clinically meaningful population health benefits, including reductions of 23.678 person-years per 100 patients of prediabetes and 25.963 person-years of T2D per 100 patient, along with additional reductions in several obesity-related comorbidities in the non-T2D population. The model also suggested improvements in non-diabetes comorbidities, including reductions in time spent with gout (14.662 and 20.347 patient-years per 100 patient lifetime in the non-T2D and T2D cohorts, respectively) and OSA (11.441 and 1.633 patient-years per 100 patient lifetime, respectively).

Overall, compared with D&E alone, semaglutide 2.4 mg was projected to prevent several comorbidities through long-term weight management, resulting in both health gains and downstream cost offset.

The cost offsets observed in our model were driven mainly by T2D microvascular complications, gout, OSA, and CV events in the non-T2D cohort (-¥32 664, -¥18 478, -¥10 478, and -¥13 093 per patient lifetime, respectively), and by gout and CV events in the T2D cohort (-¥32 666 and −¥15 904 per patient lifetime). In Japan, where Asian patients exhibit higher T2D risk and gout prevalence is high, relative to Europeans at the same BMI,^21–23^ preventing these complications addresses a significant unmet need.

In the previous CEA of semaglutide 2.4 mg conducted by C2H in Japan, retreatment was not considered.^10^ This modeling approach is consistent with several international HTA evaluations of obesity medicines, approving treatment durations up to 20 years for reimbursement.^24^ NICE recommends semaglutide for weight management for up to 2 years, and CDA-AMC’s reimbursement review similarly assesses semaglutide within an HTA framework focused on a single, continuous course of treatment strategy rather than repeated on/off cycles.^25,26^ This was primarily because the CEA was undertaken shortly after the product’s market launch, before real-world treatment patterns could be estimated, and because retreatment had not been investigated in clinical trials, leaving the effect of reinitiation uncertain. Furthermore, earlier evaluations by NICE and HAS did not incorporate retreatment in their economic models,^26,27^ and the Japanese HTA largely followed these international approaches.

However, as demonstrated in the present analysis, models that do not account for retreatment are likely to underestimate the lifetime benefits of semaglutide 2.4 mg. Differences in model structure and assumptions between the previous CEA and the present study therefore led to substantial divergence in their cost-effectiveness estimates. When evaluating cost-effectiveness in the context of real-world clinical practice, the assumptions adopted in this study are likely to provide a more realistic representation of long-term treatment patterns and cost-effectiveness of semaglutide 2.4 mg in Japan.

Sensitivity analyses indicated that conclusions were primarily influenced by discounting and long-term weight trajectory assumptions. In one-way sensitivity analysis, the ICER remained within ¥4.30 million to 6.30 million/QALY in the non-T2D cohort and ¥5.74 million to 8.44 million/QALY in the T2D cohort when key parameters were varied, and no plausible variation changed the direction of incremental costs and QALYs. PSA results were consistent with these findings, with simulations clustering in the northeast quadrant and CEACs, showing increasing probability of cost-effectiveness at higher willingness-to-pay thresholds.

This study is the first to evaluate the cost-effectiveness of semaglutide 2.4 mg with explicit incorporation of retreatment, reflecting the relapsing nature of obesity disease in Japan. By building on a modeling framework previously assessed and validated within the Japanese CEA context, the analysis maintains methodological consistency with national evaluation standards while filling a major gap in prior assessments. The inclusion of detailed clinical, economic, and retreatment-specific assumptions provides a more realistic representation of long-term treatment trajectories in real-world practice.

This study has several limitations. First, retreatment efficacy has not been evaluated in clinical trials, and no relevant published literature is currently available. In the present analysis, the effect size of the retreatment was based on the effect of the initial treatment course observed in the clinical trials, and this assumption was reviewed and validated by a specialist physician in obesity disease who is a board member of the Japanese Society for the Study of Obesity and has clinical experience in endocrinology and metabolism in Japan. Because retreatment effects were applied before outcomes fully returned to baseline, it is theoretically possible that BMI could decline further with repeated courses. Some more recent trials permitted prior GLP-1 exposure beyond a 6- to 12-month washout period, partially capturing retreatment effects, whereas such exposure was likely minimal in earlier semaglutide trials such as STEP 6. As natural weight regain was incorporated in the model, we implicitly assumed that BMI values were constrained within clinically plausible range for people with obesity (ie, BMI ≥27).^9^

Second, the dropout approach used in the original C2H model was modified to better reflect discontinuation patterns observed in clinical practice based on clinical expert input.^10^ However, long-term discontinuation rates for semaglutide 2.4 mg and the impact of retreatment on discontinuation remain uncertain. As a result, the dropout assumptions used in this model are also subject to uncertainty. As evidence on long-term treatment persistence with semaglutide 2.4 mg accumulates, the dropout assumptions applied in the present model should be re-evaluated.

Finally, the study population was informed by the STEP clinical trial program, which enrolled patients with relatively severe obesity disease and specific eligibility criteria. As such, the findings of this analysis may not be fully generalizable to the broader Japanese population with obesity disease, and interpretation should be limited to patient populations with clinical characteristics comparable to those represented in STEP and eligible under the Japanese treatment guidelines. In addition, some model inputs were derived from non-Japanese data sources. In particular, HbA1c progression among patients with T2D was based on the UKPDS 68,^18^ and baseline complication-free utilities were estimated using Health Survey for England data.^19^ Both inputs were retained from the original CEA model for consistency.^10^

Despite these limitations, this study provides novel insights as the first evaluation of retreatment with semaglutide 2.4 mg in Japan. The use of a modeling framework previously applied and validated within the national HTA context enhances the credibility and relevance of the findings. In addition, the use of Japanese-specific utility values where available and Japanese real-world claims data for key complication costs further supports the relevance of the analysis to the Japanese healthcare setting.

As evidence regarding the treatment effect of retreatment and the long-term discontinuation of semaglutide 2.4 mg remains limited, further validation will be required as additional clinical and real-world data become available. Future studies should also evaluate the broader societal and economic implications of long-term obesity disease management, including productivity effects, caregiver burden, and other value elements beyond healthcare costs that are not captured in conventional HTA, as conceptualized by the ISPOR value flower framework.^28^ Moreover, research involving more diverse Japanese populations, beyond the trial informed cohorts used in the present analysis, will be important to ensure generalizability across demographic and clinical characteristics. Finally, future evaluations should investigate how alternative policy structures, with greater flexibility in initiation, retreatment, and off-treatment intervals, may affect long-term clinical outcomes and overall economic value.

## CONCLUSIONS

Semaglutide 2.4 mg is a cost-effective treatment for obesity disease in Japan, offering substantial long-term clinical and economic benefits under clinically plausible retreatment assumptions. The analysis demonstrates that allowing for retreatment significantly enhances the long-term value of semaglutide 2.4 mg, with improved clinical outcomes and more favorable cost-effectiveness estimates compared with no retreatment. While several modeling assumptions were required to evaluate long-term outcomes in a specific patient population, these findings highlight the importance of considering clinically plausible long-term treatment pathways when assessing the value of obesity medicines in Japan.

### Disclosures

Yuta Kamada and Shogo Wada are employees of Novo Nordisk Pharma Ltd. Hiroyuki Matsuda and Yawen Dai are employees of IQVIA Solutions Japan G.K.; IQVIA Solutions Japan G.K. received consulting fees from Novo Nordisk Pharma Ltd. for this project.

## Supplementary Material

Online Supplementary Material

## Data Availability

The datasets generated during and/or analyzed during the current study are available from the corresponding author on reasonable request.
