# Supplementary material for: Cost-Effectiveness of Semaglutide 2.4 mg for Obesity Disease Management in Japan: A Lifetime Economic Modeling Study Incorporating Retreatment Scenarios
Source: J Health Econ Outcomes Res. 2026 Jun 30;13(1):288–96. doi: 10.36469/001c.162614 (PMC13327695; doi:10.36469/001c.162614)
Supplement: Online Supplementary Material [file jheor_2026_13_1_162614_351933.pdf]

Journal of Health Economics and Outcomes Research

Online Supplementary Material

Cost-Effectiveness of Semaglutide 2.4 mg for Obesity Disease Management in Japan: A Lifetime Economic Modeling Study Incorporating Retreatment Scenarios. *JHEOR*. 2026;13(1):288-296.
doi:10.36469/jheor.2026.162614

Table S1: Parameters – Utility, Costs, Health Care Resource Use and Disease-Specific Mortality

Figure S1: Tornado Diagrams (One-Way Sensitivity Analysis)

Figure S2. Cost-Effectiveness Acceptability Curve for Semaglutide 2.4 mg vs Diet and Exercise

This supplementary material has been provided by the authors to give readers additional information about their work.

This is an open-access article distributed under the terms of the Creative Commons Attribution 4.0 International License (CCBY-4.0). View this license's legal deed at http://creativecommons.org/licenses/by/4.0 and legal code at http://creativecommons.org/licenses/by/4.0/legalcode for more information.

Table S1. Parameters–Utility, Costs, Health Care Resource Use ·and Disease-Specific Mortality

Variable	Value	Description and Reference
Baseline utility	0.874	Luah et al, 2024[1]
Health state disutilities		
T2D	-0.046	Shiroiwa et al 2021[2]
Post-ACS	-0.04	Kodera et al 2018[3]
OSA	-0.047	Kawakami et al 2020[4]
Cancer, decompensated liver cirrhosis	-0.084	Shiroiwa et al 2021[2]
Post stroke	-0.24	Kodera et al 2018[3]
Pre-diabetes	0	Assumption
Gout	-0.012	Shiroiwa et al 2021[2]
Event disutility		
Bariatric surgery	-0.2	Campbell et al 2010[5], Shiroiwa et al 2021[2]
ACS	-0.073	Shiroiwa et al 2021[2]
Musculoskeletal	-0.023	Sullivan et al, 2011[6]
Stroke	-0.265	Shiroiwa et al 2021[2]
TIA	-0.033	Sullivan et al 2011[6]
Liver transplant	-0.07	Lim et al 2015[7]
Adverse event disutility		
Disutility severe gastrointestinal events	-0.001	NICE STA [TA494] [8]
Disutility non-severe hypoglycemia	-0.006	Foos et al 2018[9]
Disutility severe hypoglycemia	-0.015	Same as above
Obesity treatment costs (95% CI)		
Semaglutide 2.4 mg	¥10,096	Cost per day × 7= cost per weekly injection
Semaglutide 1.7 mg	¥7,429	Same as above
Semaglutide 1.0 mg	¥5,557	Same as above
Semaglutide 0.5 mg	¥3,009	Same as above
Semaglutide 0.25 mg	¥1,764	Same as above
Monitoring costs for obesity, annual	¥24,908 (24,671-25,145)	IQVIA claims database
Diet & exercise (if in addition to	¥2,724	Same as above

Variable	Value	Description and Reference
obesity monitoring costs above)	(2,697-2,752)	
Blood pressure treatment (most used ACEi)	¥25,533 (15,960-34,467)	Same as above
T2D weighted average of insulin and oral treatments	¥58,034 (47,442-65,645)	Same as above
Costs adverse events (per event) (95% CI)		
Non-severe hypoglycemia	¥0	Glucose intake (self-treated), reimbursed ¥0
Severe hypoglycemia	¥22,189 (16,642-27,736)	IQVIA claims database
Severe gastrointestinal events	¥8,167 (275-16,058)	Same as above
STATE COSTS (annual)-exclude acute care costs related to events		
T2D microvascular complications costs (excl. treatment costs above)	¥183,427 (159,941-199,449)	Same as above
Prediabetes	¥5,663 (0-25,750)	Same as above
Post T2D (cured)	¥0	Same as above
Cancer treatment colon in first year	¥822,111 (609,320-1,017,877)	Same as above
Cancer treatment breast in first year	¥951,561 (686,552-1,209,276)	Same as above
Cancer treatment endometrial in first year	¥961,007 (394,944-1,582,851)	Same as above
Cancer treatment (average of colon, breast, endo) in follow-up year	¥158,666 (93,129-220,804)	Same as above
MI first year, excl. acute event cost	¥172,792 (45,207-330,191)	Same as above
Unstable angina first year, excl. acute event cost	¥306,849 (152,341-482,855)	Same as above
Post acute coronary syndrome (MI or Unstable angina, in year following the event)	¥224,620 (134,614-321,270)	Same as above

Variable	Value	Description and Reference
Stroke first year, excl. acute event cost	¥939,026 (652,612-1,200,263)	Same as above
TIA, first year, excl. acute event cost	¥0	Assumed to be zero due to negative incremental cost
Post-stroke (stroke and TIA, in year following the event)	¥79,481 (35,444-122,711)	IQVIA claims database
Sleep apnea cost (eg, annual management with continuous positive airway pressure)	¥125,282 (104,036-142,557)	Same as above
Decompensated liver cirrhosis in first year	¥91,524 (22,356-183,508)	Same as above
Hepatocellular carcinoma in first year	¥115,211 (25,861-221,715)	Same as above
NAFLD in follow-up year	¥0	Assumed to be zero due to negative incremental cost
Gout, follow-up year	¥209,404 (19,024-526,154)	IQVIA claims database
Decompensated liver cirrhosis in follow-up year	¥59,393 (1,906-136,930)	Same as above
HCC in follow-up year	¥226,347 (34,233-456,631)	Same as above
Acute care costs (per event) (95% CI)		
MI nonfatal event	¥1,148,094 (993,830-1,266,707)	IQVIA claims database
MI fatal event	¥1,844,271 (95,128-4,725,732)	Same as above
Unstable angina nonfatal event	¥1,194,538 (627,583-1,914,847)	Same as above
Unstable angina fatal event	¥1,946,090 (84,839-5,920,989)	Same as above
Stroke nonfatal event	¥1,319,377 (1,073,294-1,561,398)	Same as above
Stroke fatal event	¥2,121,460	Same as above

Variable	Value	Description and Reference
	(119,598-5,710,902)	
TIA event	¥475,682 (229,907-813,022)	Same as above
Bariatric surgery, preoperative management	¥14,235 (5,399-23,071)	IQVIA claims database
Gastric bypass procedure	¥3,252,411 (2,386,332-8,232,935)	Calculated based on sleeve gastrectomy, which is the most commonly performed bariatric surgery for severe obesity in Japan
Laparoscopic banding procedure	¥3,252,411 (2,386,332-8,232,935)	Same as above
Sleeve gastrectomy procedure	¥3,252,411 (2,439,309-8,303,570)	IQVIA claims database
Bariatric surgery, post-operative follow-up	¥323,345 (102,235-544,454)	Same as above
Bariatric surgery, complications (leaks)	¥142,951 (84,817-201,084)	Same as above
Bariatric surgery, TOTAL nonfatal	¥3,592,335 (2,542,597-8,866,744)	Calculated as the weighted average of the costs of gastric bypass, laparoscopic banding, and sleeve gastrectomy procedures.
Bariatric surgery, TOTAL fatal	¥3,592,335 (2,542,597-8,866,744)	Assumed to be the same as bariatric surgery, nonfatal
Knee replacement, nonfatal	¥2,593,940 (1,667,041-4,578,678)	IQVIA claims database
Knee replacement, fatal	¥2,593,940 (1,667,041-4,578,678)	Assumed to be the same cost for nonfatal and fatal
Liver transplant	¥15,086,969 (7,489,353-22,468,058)	Kawaguchi et al 2020[10]
Disease specific probabilities of death		
Case fatality MI (% per event)	38.80	Rumana et al 2014[11]
Case fatality angina (% per event)	38.80	Rumana et al 2014[11]

Variable	Value	Description and Reference
Case fatality stroke (% per event)	13.60	Takashima et al 2020[12]
Post-ACS (HR applied to general mortality)	1.3	Johansson et al, 2017[13] (The relative risk for all-cause death and cardiovascular outcomes (recurrent MI, cardiovascular death) was at least 30% higher than that in a general reference population at both 1-3 years and 3-5 years after MI)
Post-stroke (HR applied to general mortality)	2	Brammås et al, 2013[14] (The 1-year mortality was 36.5% for AMI complicated by ischemic stroke and 18.3% for AMI without stroke, hence 2X higher)
Case fatality knee replacement (%)	0.36	Sinclair et al 2021[15]
Colorectal cancer (% in year of onset)	33.74	Cancer Registry Data 2019[16]; calculated by dividing the 2019 crude mortality rate for colorectal cancer by the crude incidence rate.
Breast cancer (% in year of onset)	15.60	Same as above; calculated by dividing the 2019 crude mortality rate for breast cancer by the crude incidence rate among females.
Endometrial cancer (% in year of onset)	14.86	Same as above; calculated by dividing the 2019 crude mortality rate for endometrial cancer by the crude incidence rate.

Variable	Value	Description and Reference
Decompensated liver cirrhosis (%)	43.30	Fujiyama et al, 2021[17]; complement of the 5-year cumulative survival rate in patients with decompensated liver cirrhosis.
HCC (%)	69.30	Same as above
Case fatality for liver transplant (%)	6.80	Gong et al, 2020[18]; mortality rate during the initial hospitalization for liver transplantation.
Cancer (% in years 2+ from onset)	7.60	Cancer Registry Data 2019[16]; derived from the 5-year relative survival rate for localized stage cancer.

Abbreviations: ACS, acute coronary syndrome; HCC, hepatocellular carcinoma; HCRU, healthcare resource use; HR, hazard ratio; MI, myocardial infarction; NAFLD, nonalcoholic fatty liver disease; NASH, nonalcoholic steatohepatitis; OSA, obstructive sleep apnea, QOL, quality of life; T2D, type 2 diabetes; TIA, transient ischemic attack.

Figure S1. Tornado Diagrams (One-Way Sensitivity Analysis)

Subgroup: non-T2D at baseline

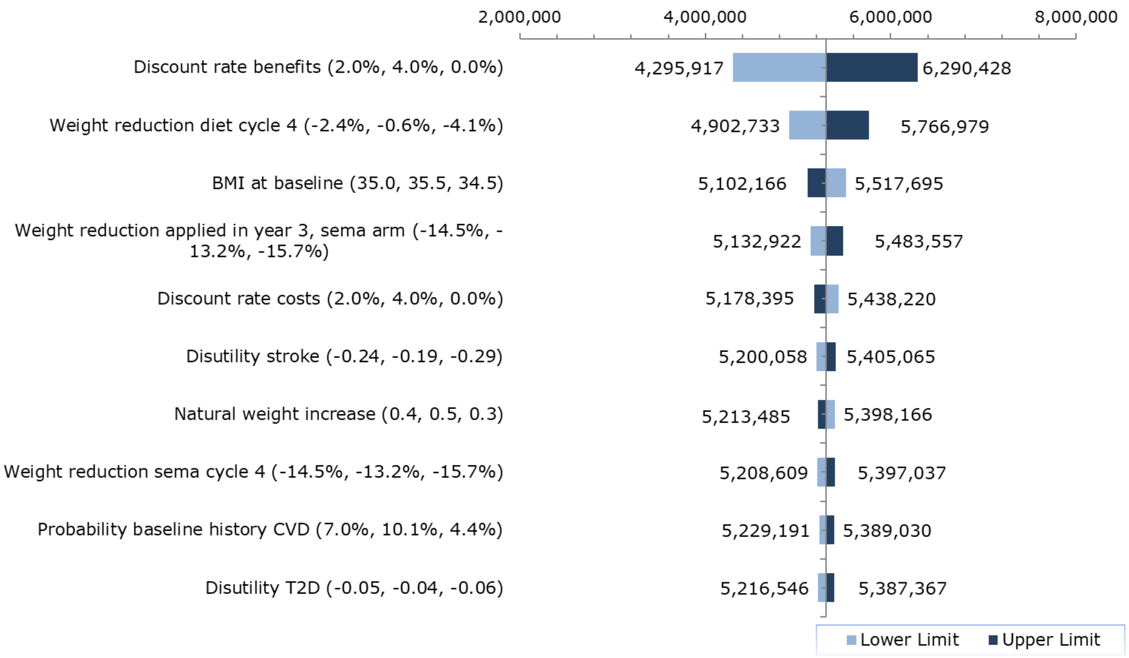

Subgroup: T2D at baseline

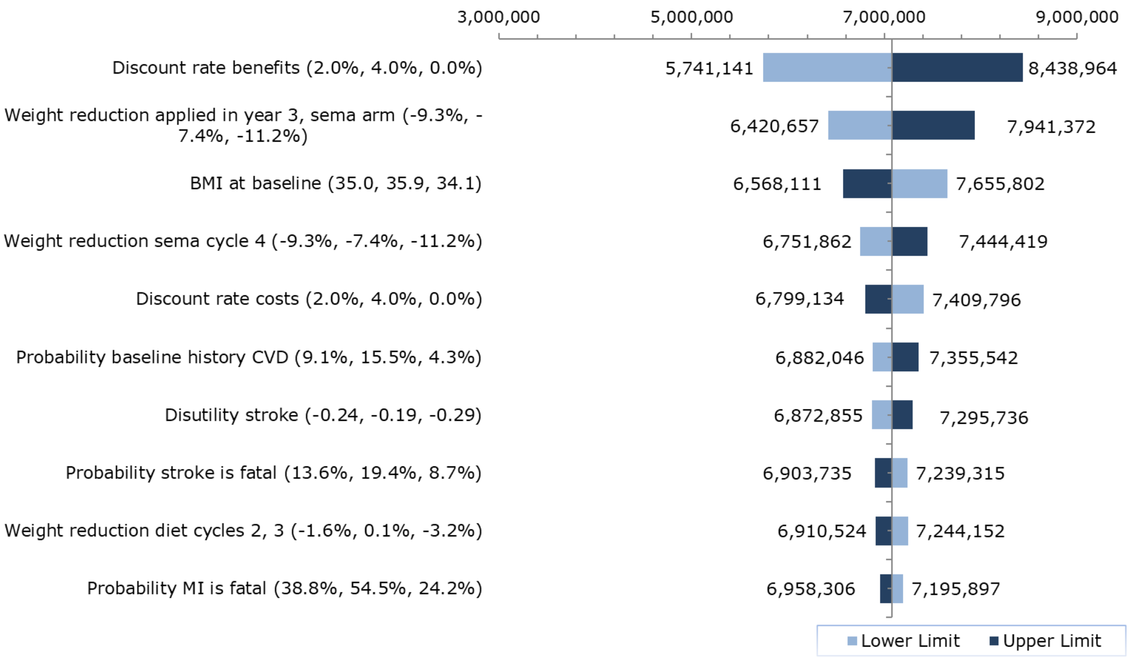

Abbreviations: BMI, body mass index; CVD, cardiovascular disease; MI, myocardial infarction; T2D, type 2 diabetes.

Note: Horizontal bar chart. Center axis is Base Case ICER. Values in each parameter means base case, upper value, and lower value from left.

Figure S2. Cost-Effectiveness Acceptability Curve for Semaglutide 2.4 mg vs Diet and Exercise

Subgroup: Non-T2D at baseline

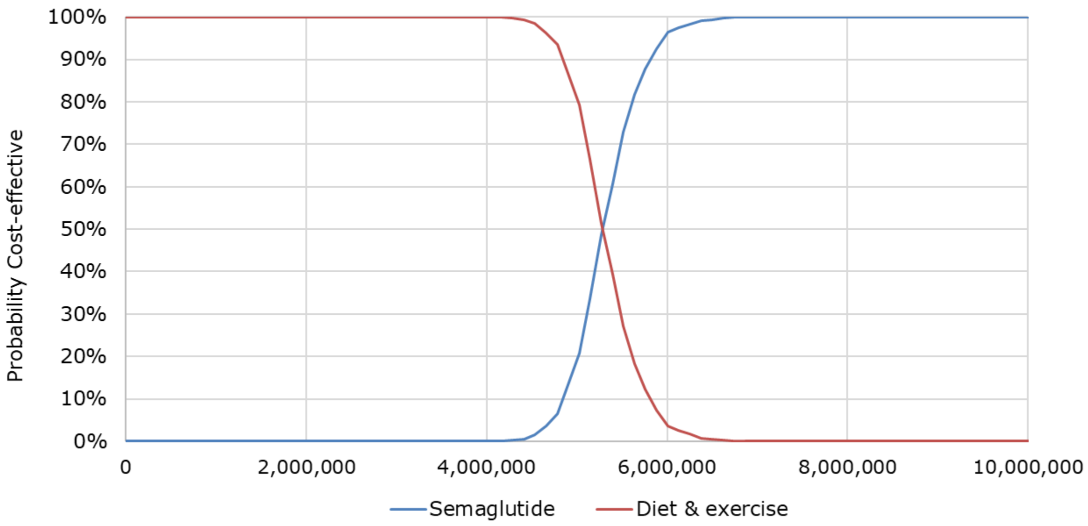

Subgroup: T2D at baseline

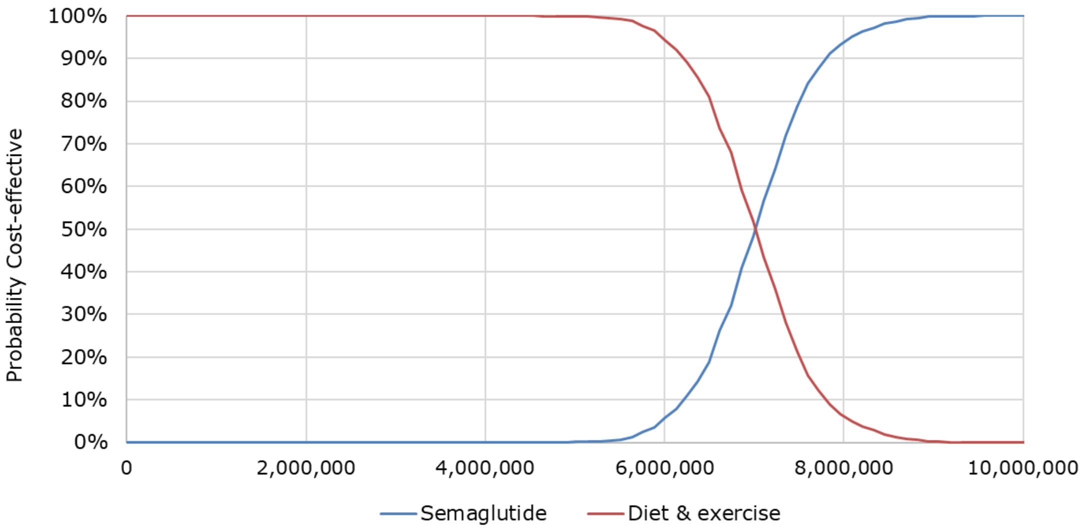

Note: Line graph. X-axis: willingness-to-pay threshold (0-¥10M). Y-axis: Probability Cost-Effective (%).

REFERENCES

1. Luah XW, Holst-Hansen T, Lubker C. The association between body mass index and health-related quality of life in the 2017 and 2018 health survey of England data: a cross-sectional observational analysis. *Diabetes Obes Metab*. 2024;26(6):2318-2328. doi:10.1111/dom.15546
2. Shiroiwa T, Noto S, Fukuda T. Japanese Population Norms of EQ-5D-5L and Health Utilities Index Mark 3: Disutility Catalog by Disease and Symptom in Community Settings. *Value Health*. 2021;24(8):1193-1202. doi:10.1016/j.jval.2021.03.010
3. Kodera S, Morita H, Kiyosue A, Ando J, Takura T, Komuro I. Cost-effectiveness of PCSK9 inhibitor plus statin in patients with triple-vessel coronary artery disease in Japan. *Circ J*. 2018;82(10):2602-2608. doi:10.1253/circj.CJ-17-1455
4. Kawakami H, Saito M, Kodera S, et al. Cost-effectiveness of obstructive sleep apnea screening and treatment before catheter ablation for symptomatic atrial fibrillation. *Circ Rep*. 2020;2(9):507-516. doi:10.1253/circrep.CR-20-0074
5. Campbell J, McGarry LA, Shikora SA, Hale BC, Lee JT, Weinstein MC. Cost-effectiveness of laparoscopic gastric banding and bypass for morbid obesity. *Am J Manag Care*. 2010;16(7):e174-187.
6. Sullivan PW, Slejko JF, Sculpher MJ, Ghushchyan V. Catalogue of EQ-5D scores for the United Kingdom. *Med Decision Making*. 2011;31(6):800-804.
7. Lim KC, Wang VW, Siddiqui FJ, et al Cost-effectiveness analysis of liver resection versus transplantation for early hepatocellular carcinoma within the Milan criteria. *Hepatology*. 2015;61(1):227-37. doi:10.1002/hep.27135
8. National Institute for Health and Care Excellence. Naltrexone–bupropion for managing overweight and obesity. https://www.nice.org.uk/guidance/ta494
9. Foos V, McEwan P. Conversion of hypoglycemia utility decrements from categorical units reflecting event history into event specific disutility scores applicable to diabetes decision models. *Value Health*. 2018;21:S223. doi:10.1016/j.jval.2018.04.1506
10. Kawaguchi I, Chayama K, Gonzalez YS, et al. A cost-effectiveness analysis of glecaprevir/pibrentasvir versus existing direct-acting antivirals to treat chronic hepatitis C in Japan. *Adv Ther*. 2020;37(1):457-476. doi:10.1007/s12325-019-01166-3
11. Rumana N, Kita Y, Turin TC, et al. Acute Case-fatality rates of stroke and acute myocardial infarction in a Japanese population: Takashima Stroke and AMI Registry, 1989–2005. *Int J Stroke*. 2014;9(SA100):69-75. doi:10.1111/ijs.12288
12. Takashima N, Arima H, Kita Y, et al Two-year recurrence after first-ever stroke in a general population of 1.4 million Japanese patients- The Shiga Stroke and Heart Attack

Registry Study. *Circ J*. 2020;84(6):943-948. doi:10.1253/circj.CJ-20-0024
13. Johansson S, Rosengren A, Young K, Jennings E. Mortality and morbidity trends after the first year in survivors of acute myocardial infarction: a systematic review. *BMC Cardiovasc Disord*. 2017;17(1):53.
14. Brammås A, Jakobsson S, Ulvenstam A, Mooe T. Mortality after ischemic stroke in patients with acute myocardial infarction: predictors and trends over time in Sweden. *Stroke*. 2013:STROKEAHA. 113.001434.
15. Sinclair ST, Orr MN, Rothfusz CA, Klika AK, McLaughlin JP, Piuzzi NS. Understanding the 30-day mortality burden after revision total knee arthroplasty. *Arthroplasty Today*. 2021;11:205-211. doi:https://doi.org/10.1016/j.artd.2021.08.019
16. National Cancer Center Japan. Cancer Registry Data. https://ganjoho.jp/reg_stat/statistics/stat/cancer/1_all.html
17. Fujiyama S, Akuta N, Sezaki H, et al Mortality rates and risk factors in 1412 Japanese patients with decompensated hepatitis C virus-related cirrhosis: a retrospective long-term cohort study. *BMC Gastroenterol*. 2021;21(1):189. doi:10.1186/s12876-021-01770-0
18. Gong N, Jia C, Huang H, Liu J, Huang X, Wan Q. Predictors of mortality during initial liver transplant hospitalization and investigation of causes of death. *Ann Transplant*. 2020;25:e926020. doi:10.12659/aot.926020
